# A framework for evaluating less-than-lifetime exposures: advancing toxicological risk assessment for drinking water quality

**DOI:** 10.1007/s00204-025-04061-9

**Published:** 2025-05-25

**Authors:** Sanah Majid, Astrid Reus, Renske Hoondert, Mirjam Blokker, Amitosh Dash, Corine Houtman, Merijn Schriks, Milou M. L. Dingemans

**Affiliations:** 1 KWR Water Research Institute, Nieuwegein, The Netherlands; 2 Het Waterlaboratorium, Haarlem, The Netherlands; 3 Amsterdam Institute for Life and Environment (A-LIFE), Vrije Universiteit Amsterdam, Amsterdam, The Netherlands; 4 Vitens NV, Water Company, Zwolle, The Netherlands; 5 Institute for Risk Assessment Sciences, Utrecht University, Utrecht, The Netherlands

**Keywords:** Chemical contaminants, Drinking water safety, Health risk assessment, Risk-based monitoring, Public health

## Abstract

The increasing production and use of chemicals leads to a higher number of contaminants in (drinking) water sources, which poses significant challenges to those responsible for water quality. An important task for water companies and regulators is to ensure that (drinking) water quality does not compromise public health and confidence in water safety. A key component of this effort is toxicological risk assessment, which evaluates the safety of chemicals that may be present in water systems. Although there has been considerable progress in risk assessment methods, there is currently a lack of a clear approach to the risk assessment of chemicals in (drinking) water for which concentrations vary over time. In the current study, methods for the assessment of less than lifetime exposure (LTL) are presented that are particularly applicable for substances with time-varying concentrations in (drinking) water. The presented framework is a decision tree that helps experts to decide whether a measured or predicted LTL exposure to a chemical can lead to adverse health effects. To develop these methods, we used existing knowledge and incorporated new developments in toxicological risk assessment. A case study illustrates the application of the proposed approaches in a realistic scenario. The method can be used by risk assessors as a tool to improve the understanding of human exposure to contaminants in (drinking) water and to inform risk-based monitoring. In addition, it enables customised investigations and responses that lead to a more nuanced view of water quality and its impact on public health.

**Supplementary Information:**

The online version contains supplementary material available at 10.1007/s00204-025-04061-9.

## Introduction

Chemical contaminants enter the environment through various sources, including agriculture, domestic use, and industries (Golovko et al. 2021; Li et al. 2024). Once released, these chemicals disperse into the air, water, soil, and food, potentially exposing humans through inhalation, ingestion, or dermal contact, leading to adverse health effects. The rising detection of chemicals in water bodies has increased concerns over drinking water safety. This increased detection is attributed to higher chemical production, prolonged dry periods due to climate change, and advancements in detection technologies (Sjerps et al. 2017; Béen et al. 2021). In addition, human activities, such as agriculture, aviation, and industrial operations, also impact groundwater storage and dynamics, resulting in a decline in water quality (Zhao et al. 2024). Excessive groundwater extraction further disturbs the natural flow patterns of water and mobilises contaminants like nitrates, heavy metals, and pesticides into groundwater systems, thereby increasing health risks and ecological damage (Wu et al. 2019; Hou et al. 2023). Heavy rainfall events further intensify this issue by increasing surface runoff which transports contaminants into recharge zones and further deteriorates ground water quality (Hou et al. 2023). In addition, inadequate waste management during wastewater treatment can introduce additional contaminants into aquatic systems. For example, improperly managed sludge from treatment processes can leach organic and inorganic contaminants, including heavy metals, pharmaceuticals, and other toxic substances, into groundwater and surface waters (Morin-Crini et al. 2022). Populations at higher health risk are the ones that are more sensitive or have a relatively high exposure to harmful contaminants (e.g., embryos or foetuses exposed via mother, infants, children, elderly and patients). A health risk assessment is an important process aimed at estimating the nature and probability of adverse health effects in individuals potentially exposed to chemicals in contaminated environmental mediums, either presently or in the future.

In the European Union (EU), risks of chemical substances are generally assessed separately across different sectors, such as pesticides, pharmaceuticals, and industrial chemicals, which results in varied assessment methods. This variation arises mainly from differences in the information required by each regulatory framework. To harmonise this process, the EU has proposed the “one substance—one assessment” approach under the Green Deal and Zero - Pollution targets (van Dijk et al. 2021; Escher et al. 2022). Evaluation of health risks of (drinking) water contaminants depends on factors such as the type and concentration of the contaminant, individual sensitivity, quantity of water consumed, frequency and duration of exposure. Exposure duration is particularly crucial in assessing health risks (Schwela 2024; de Oliveira et al. 2021). Generally, risk assessments assume constant, lifetime (chronic) exposure to contaminants, including those for drinking water, as outlined in the WHO Guidelines for Drinking-Water Quality (WHO 2022a, b, c). However, real-world exposure patterns are often more variable (Amachree et al. 2013, 2014). In particular, health effects of less-than-lifetime (sub-chronic) exposure may differ from those of chronic exposure. Therefore, evaluating short-term exposure risks requires specific approaches.

**Fig. 1:**
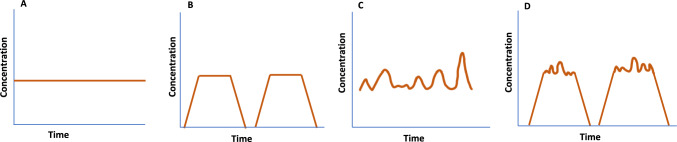
Different chemical exposure categories: **A** continuous, **B** intermittent, **C** fluctuating exposure and **D** combination of intermittent and fluctuating exposure (adapted from: Geraets et al. 2016)

In the present study, the term “LTL risk assessment” refers to evaluating the risk posed by chemicals to human health, exposed for a period lasting less than a lifetime, which is generally considered to be 70 years. These exposures include intermittent (non-continuous/regular) or fluctuating (irregular) exposures, occurring for acute/very short (1–14 days), short (more than 14 days to 1 year) or intermediate durations (more than 1 year to 7 years) (see Fig. 1), as adapted from the framework for LTL risk assessment developed by Felter et al. (2011). Intermittent exposure occurs in non-continuous patterns, with repeated exposure cycles that influence toxicity outcomes based on duration, frequency, and intensity (MacPhail 2001). Such exposures are required to be separated by a sufficiently long duration so that the steady state of exposure is not maintained, i.e. equilibrium of concentrations between the external environment and the body compartments is not achieved. In these cases, accurate predictions of bioaccumulation and toxicity effects may be challenging to ascertain.

Studies have shown that intermittent exposure to various substances can significantly impact physiological parameters and mortality rates in animals, which emphasises the need for human health risk assessment frameworks for short and intermittent duration exposures (Matisoff et al. 1996; Panter et al. 2000; Amachree et al. 2014). The previous framework focused on non-carcinogenic effects of short- and intermittent-duration exposures, emphasizing the need for improved dose averaging and exposure assessment methods (Haber et al. 2016). One key aspect is the necessity for the exposure duration to surpass the time required for the system to reach equilibrium (Undeman et al. 2009). This prerequisite emphasises the significance of allowing sufficient time for the chemical to distribute and accumulate within the organism until an equilibrium is achieved. Such irregular exposure patterns may potentially alter the body’s response mechanism in several ways, such as impairing the ability of the body to effectively deal with toxins when associated processes are overwhelmed or to be unable to function optimally. In addition, irregular exposures can lead to periods of high chemical concentration in the body, which may reach peak levels during exposure peaks, resulting in acute toxic effects (Checkoway et al. 2015; Allegra et al. 2019).

The current study aimed to develop a general framework for assessing chemical exposures via (drinking) water that correspond to LTL exposure scenarios. The proposed approach provides a tailored method for assessing chemical water quality for better understanding and management of temporary chemical exposures for more accurate health risk assessments.

## Methods

Risk assessment methods and other relevant information for the LTL risk assessment were compiled from a scientific literature search. Peer-reviewed literature and (inter)national (meta) databases and websites or reports published by renowned institutes and authorities for human health protection were searched, including those by the World Health Organisation (WHO), US Environmental Protection Agency (USEPA), European Food and Safety Authority (EFSA), European Chemical Agency (ECHA), Public Health England (PHE), and the Dutch National Institute for Public Health and the Environment (RIVM). To ensure a thorough and relevant search, specific search terms were used. These terms included “short-term exposure + chemicals + oral”, “risk assessment + less-than-lifetime”, “sub-chronic toxicity + health risk”, “low-level chemicals + drinking water” + short-term exposure + intermediate exposures”. The selection criteria were based on their relevance to LTL risk assessment and applicability to drinking water scenarios. The literature obtained from these searches was summarised and contextualised in relation to (drinking) water. Based on the obtained information and expert insights, a framework for LTL risk assessment was developed. This framework was then practically applied in a case study that describes a scenario of exposure to lead (Pb) via drinking water to demonstrate the practical application of the recommended framework in a drinking water context. Lead was chosen for the case study due to its regulatory focus, relevance to drinking water safety, and significant public concern.

## Results literature review

### Health-based guidance values (HBGVs) in chemical risk assessment

Health-based guidance values (HBGVs) are science-based recommendations that define the maximum allowable exposure to chemical substances exhibiting a threshold of toxicity (Niemann et al. 2023). These values play a key role in risk assessment, as they help in establishing safe exposure limits for substances in the environment, food, or water to ensure that exposure remains within levels that are unlikely to pose appreciable health risks (Rose 2015; EFSA 2021). The process of deriving HBGVs involves a comprehensive review of scientific data, considering factors such as, toxicity, exposure durations and uncertainties (see Tables 1 and 2). The conventional approach to establishing HBGVs involves determining the dose–response relationship for adverse effects and identifying a reference point (RP), such as no-observed-adverse-effect level (NOAEL), the lowest-observed-adverse-effect level (LOAEL), or the lower confidence limit of the benchmark dose (BMDL) when applicable (EFSA 2021). These RPs are based on the most sensitive endpoint relevant to humans and used to establish the HBGV, which is adjusted using safety factors (uncertainty factors) to account for inter-species and inter-individual variability, as well as gaps in evidence (Rose 2015; EFSA 2021). An example of commonly used HBGV is the acceptable daily intake (ADI) (expressed as mg/kg/day), which estimates the safe intake levels of a chemical substance (e.g., food additives, pesticide residues and veterinary drugs) via food or drinking water (Herrman and Younes 1999; EFSA 2021; Gray, 2023). In addition, reference doses (RfDs) and tolerable intakes (TIs), such as tolerable daily intakes (TDIs) and tolerable weekly intakes (TWIs), are the other commonly used HBGVs.

**Table 1:** Different types of toxicological studies used for testing chemicals, including pesticides or active substances for crop protection and industrial chemicals

Type of study	Duration	Animal species	OECD test guideline
Long-term	Major portion of lifespan, including: 24 months exposure durations (18 months for specific mice strains)	Rodents (rats and mice)	OECD TG 451, OECD TG 453
Chronic	Long duration (12 months) or major portion of lifespan, (18 or 24 months) or shorter (6 or 9 months)	(mainly) Rodents (rats and mice)	OECD TG 452, OECD TG 453
	9 months	Non-rodents (dog, beagle; other species: swine, mini-pigs)	OECD TG 452 combined with OECD TG 409, with appropriate modifications outlined in OECD Guidance Document No. 116
Sub-chronic	Part of lifespan Intermediate duration (90 days)	Rodents (preferably rat) Non-rodents (dog, beagle; other species: swine, mini-pigs)	OECD TG 408 OECD TG 409
	Short-term (sub-acute) (28 days)	Rodents (preferably rat)	OECD TG 407
Acute	Part of lifespan	Rodents (preferably rat)	OECD TG 420, OECD TG 423, OECD TG 425

**Table 2:** Existing uncertainty factors (UFs) used for deriving HBGV (Adapted from: Bercu et al. 2021; ECHA 2012c)

Source of uncertainty	Default uncertainty factor
In the absence of chemical-specific data on kinetics and/or dynamics If available and relevant, remaining components, for which data are not available	Default UF = 100 (10 for inter-species variability × 10 for intra-human variability) Inter-species variability in toxicokinetics = species dependent Inter-species variability in toxicodynamics = 2.5 Intra-human variability in toxicokinetics = 3.16 Intra-human variability in toxicodynamics = 3.16
Deficiencies in the data available for the assessment	Getting additional data to improve the quality of the dataset is recommended If additional data cannot be obtained or requested, use additional justified UF on a case-by-case basis
Accounting for the absence of a no-observed-adverse-effect-level (NOAEL)	Case-by-case basis
Severity and nature of the observed effect	Case-by-case basis
Conversion factors for administration of test substances in drinking water	No recommendations for conversion factors for toxicity studies where the substance is administered in the drinking water

The evaluation process primarily relies on in vivo (animal) studies, in vitro tests, and, when available, relevant human data (including experimental and epidemiological studies), all of which are considered across different exposure durations (Goeden 2018; PHE 2019; EFSA 2021). The RfDs used by the US Environmental Protection Agency (USEPA), is an alternative to the ADI and may result in lower levels for acceptable intake due to its more detailed evaluation process. The procedure for establishing RfDs is somewhat more detailed than for ADIs and includes the use of additional modifying factors ranging from 1 to 10 (based on professional judgement) (Barnes et al. 1988; Watts and Teel 2014; retrieved from USEPA 2023). RfDs incorporate additional safety factors to account for hypersensitivity reactions and extrapolation from experimental animal data to humans (Barnes et al. 1988; Watts and Teel 2014). These have become a widely used indicator of chronic toxicity and have been established for oral and inhalation routes.

Risk assessment methodologies primarily prioritise chronic exposure duration studies, especially those assessing long-term, lifetime exposure. These studies provide the most reliable data for determining HBGVs, as they allow enough time for adverse health effects to manifest (see Table 1). Consequently, HBGVs derived from chronic exposure are also considered protective for short-term exposures (Felter et al. 2011; Geraets et al. 2016; Goeden 2018). While HBGVs primarily focus on chronic toxicity and long-term exposure, it is important to acknowledge that acute and sub-chronic effects can also play a significant role in health risks. The derivation and application of safe values for non-chronic effects may differ from those for chronic exposures, reflecting different risk profiles associated with short-term exposure scenarios. Therefore, when evaluating risks related to chemical exposures, careful consideration of the applicability of long-duration HBGVs to LTL exposure patterns is crucial. In addition to chronic exposure studies, acute exposure studies are used to determine acute reference dose (ARfD) (USEPA 2006), which may be used as a basis to develop short-term exposure values (STEV) (Leusch et al. 2020). Such short-term or acute exposure limits are particularly relevant for LTL exposure scenarios involving relatively high acute or short-term exposures of chemicals, e.g., in situations to deal with emergencies involving accidental or intentional chemical release or other catastrophic events that are single and non-repetitive. For guidance on setting an ARfD, we recommend the readers refer to the publications of Solecki et al. (2005). STEVs are not suitable for all chemicals, particularly those that present significant health risks even from low, acute exposures. For drinking water, STEVs should only be established for chemicals with an existing drinking water guideline based on acute toxicity (Leusch et al. 2020).

The World Health Organisation (WHO) has developed a method to derive short-term health-based guidelines for chemicals in case of emergency situations (WHO 2017). This approach uses available toxicity data (such as ARfD) and allocates 100% contribution to drinking water in the short term (WHO 2017). Similar methods have also been used by the USEPA and UK Water Industry Research (UKWIR). These limits can be used to assess the severity of the event, determine potential consequences, and decide what protective measures should be taken. In advance of an uncontrolled release, these limits can also be used to assess the consequences and plan a response (DOE 2016). The decision on whether it is necessary to establish an acute reference limit for a chemical of concern is based on the hazard profile of a substance and on specific endpoints that may be particularly relevant to the effects of acute exposure. The decision as to whether the application of an ARfD is necessary should be based on the hazard profile of a chemical as well on specific endpoints which may be particularly relevant to acute effects, such as irritation of the skin, eye, mucous membrane/gastro-intestinal tract, or mucous membrane/respiratory tract) (Solecki et al. 2005; ECHA 2013).

While HBGVs are used to establish allowable levels of contaminants in water (and food) for the protection of public health, there are also chemicals (such as residues of pesticides, veterinary medicines, or biocidal products) for which the maximum residue limits (MRLs) are used as safe levels. MRL is the maximum amount of residue that is legally tolerated (regulatory limit) in or on food and animal feed when applied correctly (according to Good Agriculture Practise) and is expected to be without any health concerns. MRLs are derived for individual products. The summation of the total dose from all the products is compared with an HBGV (e.g., an ADI) to verify whether MRLs may lead to health risks or not. If no substance-specific MRL is available, a default MRL for pesticides of 0.01 mg/kg is used (EU Commission 2008).

### LTL risk assessment for (drinking) water quality

Health-based drinking water standards, referred to as guideline values (GLVs), exist for a limited number of known contaminants. These are established to ensure the safety of drinking water supplies. Main information sources on drinking water standards and guidelines are provided in Table 3. Many chemicals emerging in surface and groundwater lack drinking water GLVs due to database uncertainties, unachievably low calculated values, or detection limits. For such chemicals, a provisional or indicative guideline value may be applied for interim assessment (WHO 2022a, b, c). If LTL exposure to a chemical remains below the chronic health-based guideline value, no acute or chronic effects are expected. However, if LTL exposure exceeds the chronic guideline but stays below the acute reference value, acute effects can be ruled out, but (sub)chronic effects remain uncertain. Therefore, in a (drinking) water context, LTL risk assessment of chemicals is especially relevant when chronic health-based guideline values are exceeded for a certain period of time. For example, lead is rarely found in source waters but can enter drinking water if lead-containing plumbing materials corrode (especially if the water is high in acidity or low in mineral content). While no safe level of exposure exists for lead, the WHO has set a target value of 5 µg/L (Flora et al. 2012; Vorvolakos et al. 2016; WHO 2016, 2022a, b, c). When lead leaches from the material in contact with water, people may be (temporarily) exposed to lead for what is considered LTL, such as during the pipe replacement in the house (see case study).

**Table 3:** Information sources for drinking water standards and guidelines values (Source Baken 2018)

Legal standards	Drinking water decree (Netherlands) Regulation of materials and chemicals for drinking and hot tap water supply (Netherlands) EU Council Directives USEPA Drinking Water Regulations OEHHA California Public Health Goals	https://wetten.overheid.nl/BWBR0030111/2015-11-28 https://wetten.overheid.nl/BWBR0030279/2017-07-01 https://eur-lex.europa.eu/legal-content/EN/TXT/?uri=CELEX:01998L0083-20151027 https://www.epa.gov/dwreginfo/drinking-water-regulations https://oehha.ca.gov/water/public-health-goals-phgs
Guideline values	Australia Drinking Water Guidelines WHO Drinking-water quality guidelines USEPA Drinking Water Regulations USGS Health-Based Screening Levels Health Canada National Institute for Public Health and the Environment (Netherlands)	https://www.nhmrc.gov.au/about-us/publications/australian-drinking-water-guidelines https://www.who.int/teams/environment-climate-change-and-health/water-sanitation-and-health/water-safety-and-quality/drinking-water-quality-guidelines https://www.epa.gov/dwreginfo/drinking-water-regulations https://www.usgs.gov/programs/environmental-health-program/science/usgs-health-based-screening-levels-available-online https://www.canada.ca/en/health-canada/services/environmental-workplace-health/reports-publications/water-quality/guidelines-canadian-drinking-water-quality-summary-table.html https://rvszoeksysteem.rivm.nl/

Studies have shown that the chemical concentration in (drinking) water sources fluctuates over time, which may result in periods of higher concentrations alternating with periods of lower concentrations (Chen et al. 2009; Sjerps et al. 2017; Yang et al. 2021). For example, (short) periods of extremely low river discharge may temporarily result in higher chemical concentrations in drinking water sources (due to reduced dilution of point sources) than under normal conditions (Sjerps et al. 2017; ter Laak 2018), and thus potentially higher exposures during periods as short as three months, making LTL risk assessment also relevant for such situations and corresponding exposure scenarios. An LTL risk assessment approach can help to evaluate potential health risks from short-term exposures which are currently not often considered (Baken 2018). This can also support (drinking) water utilities as well as government and authorities in deciding if control measures need to be taken to ensure protection of human health.

### Framework for evaluating LTL exposures for (drinking) water quality

**Fig. 2:**
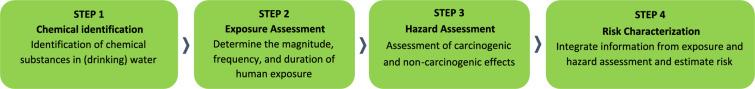
Workflow of the framework for LTL risk assessment of chemicals in (drinking) water

Felter et al. (2011), proposed a framework that provides guidance on factors that should be considered in cancer risk assessment decisions for LTL exposures based on available toxicity and exposure data. In addition, several chemical safety guidance documents for biocides, veterinary medicines, cosmetics, and industrial chemicals address the issue of fluctuating or intermittent exposures, but a clear-cut approach on dealing with such exposures, particularly in the (drinking) water context is currently lacking (EMA 2010; ECHA 2012a, b, c, 2015; SCCS 2012; Geraets et al. 2016). We thus propose a framework for assessing the health risk of chemicals in LTL exposure scenarios for drinking water quality using existing quantitative methods. The framework presented is a decision tree that helps water quality and risk experts determine whether a measured or predicted LTL exposure level may lead to adverse health effects. The framework consists of four steps (see Fig. 2). The first step is to identify the chemicals in (drinking) water relevant to the exposure scenario. Additional information could be sought for similar and/or co-occurring chemicals. The second step describes the assessment of exposure. The assessment of potential hazard of contaminants identified in drinking water is described in the third step. In this step, a method for assessing both carcinogenic and non-carcinogenic effects is presented based on the framework proposed by Felter et al (2011), in combination with principles developed by Public Health England (PHE 2019), Geraets et al. (2016), and Baken (2018), as they can be applied to chemicals in a (drinking) water context. The final step of the framework is risk characterisation. The proposed methodology attempts to address specific issues related to quantitative aspects of risk assessment for LTL exposure to chemicals in (drinking) water and provides guidance on factors that should be considered in risk assessment decisions.

#### Step 1: identification of (drinking) water contaminants

The LTL risk assessment method for (drinking) water contaminants begins with the identification of the chemicals detected in (drinking) water relevant to a particular exposure scenario and associated health risks. The most straightforward method for identifying potentially hazardous chemicals is through sampling and chemical analysis. Two types of analysis can be applied to identify chemicals in water: target compound analysis and screening (targeted, suspect or non-target screening (NTS); Hollender et al. 2019; Wang et al. 2022). In target compound analysis, known chemicals are analysed using reference standards for identification and quantification. However, target compound analysis alone is in general not sufficient because of the multitude of micropollutants that can be present in water, which urges the use of advanced approaches such as NTS. Screening allows for the simultaneous detection and characterisation of a wide range of (unknown) chemicals, including chemicals of emerging concern (CECs) (Pourchet et al. 2020; Hinnenkamp et al. 2021; Hollender et al. 2023; Dulio et al. 2024). However, screening is mostly qualitative or semi-quantitative in nature, meaning that the exact concentrations of the compounds are not assessed. From the detected compounds, a selection of those judged most relevant (based on hazard) can be made, that need to be determined quantitatively.

To accurately assess the risk, it is essential to measure how concentrations of these chemicals change over time. This requires an appropriate sampling strategy. For very short (acute) exposures, higher-frequency measurements may be needed to capture rapid fluctuations, which may occur due to short-lived pollution events or irregular contaminant releases. For these scenarios, grab sampling, where samples are taken at a specific point in time, can be used (Tadić et al. 2022). However, given the limitations of grab sampling, where multiple samples may be needed to cover temporal variations, this approach can become labour-intensive and inefficient. In such cases, an autosampler, which can automatically collect samples at regular intervals, would be a more practical alternative, providing high-frequency data with significantly less manual effort (Wilson et al. 2024). In contrast, for intermediate exposures, where contaminants may vary over extended periods compared to acute exposure, time-integrated sampling methods, such as, passive sampling, can be used (Tadić et al. 2022; Martín-García et al. 2023). This method offers the advantage of capturing temporal fluctuations in contaminant concentrations and provide a more representative view of exposure across the sampling period. Since many different types of contaminants may be present in the water distribution system, a case-by-case approach may be required for accurate qualitative and quantitative analysis of the relevant chemicals (Zulkifli et al. 2018). Parent chemical compounds can be degraded in the environment and during drinking water treatment, producing degradation products that may be of smaller, similar or higher toxicity as that of the parent compounds and the formation of degradation products should therefore be considered explicitly in exposure assessments.

#### Step 2: exposure assessment for LTL scenarios

**Fig. 3:**
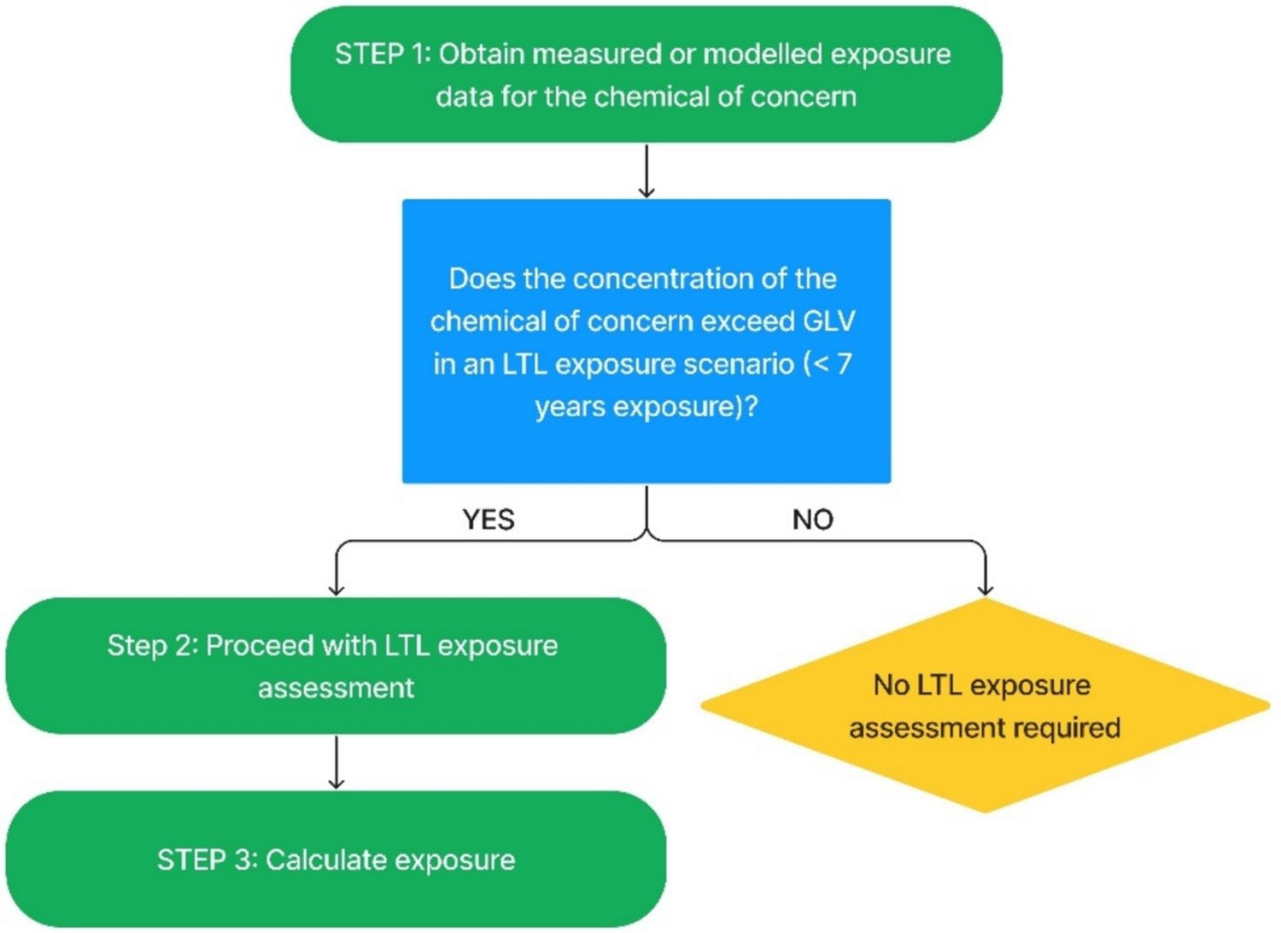
Workflow for LTL exposure assessment. Green = Start and end of steps; Blue = Process; Yellow = Decision (color figure online)

**Fig. 4:**
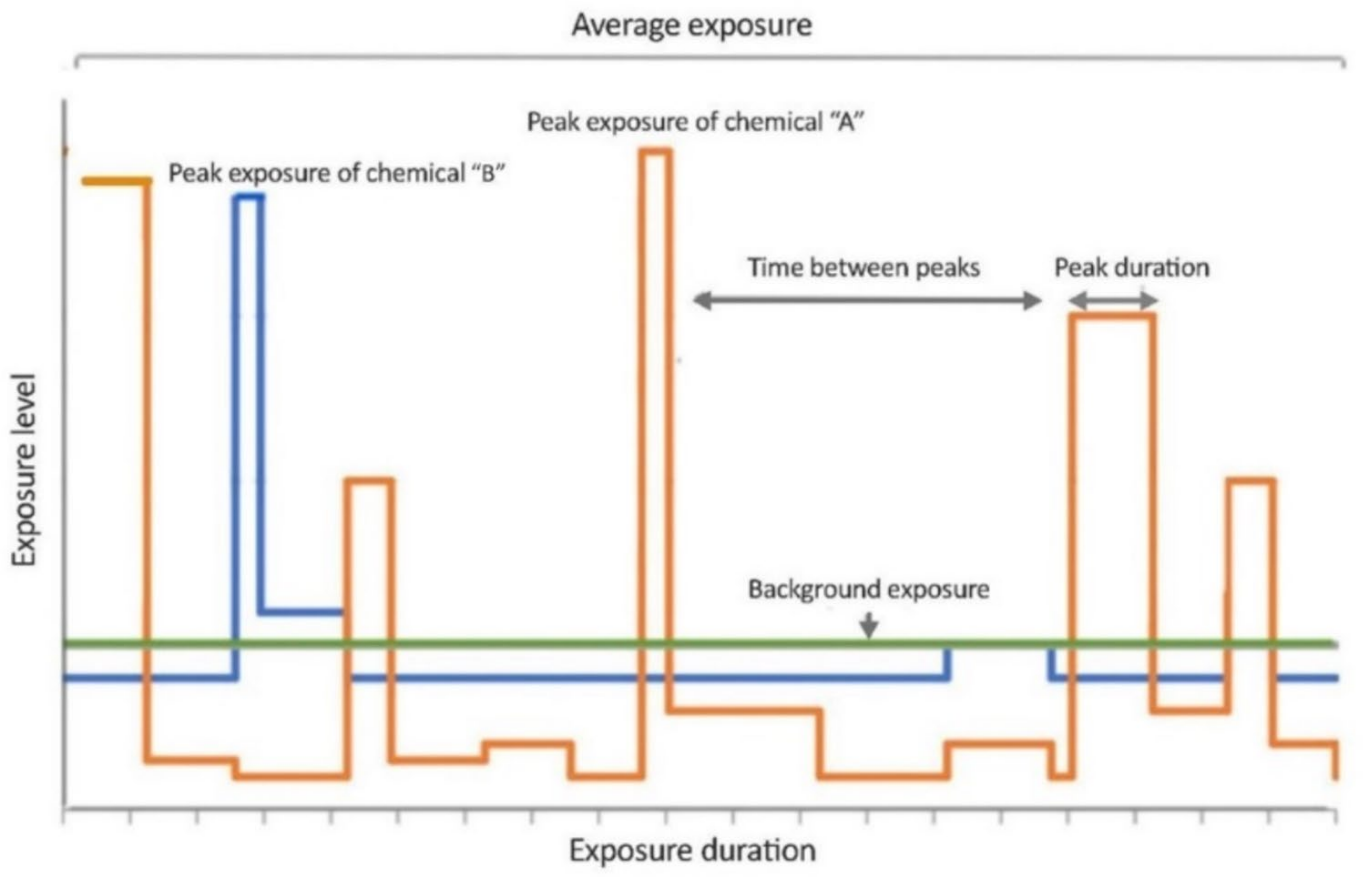
Fictional peak exposure of chemicals (orange and blue peaks) of chemicals relative to background exposure (green line) (color figure online)

Exposure assessment is a critical step in the risk assessment process and includes estimation (modelling) or measurements of chemical concentration in the relevant medium and estimation of the duration of exposure (Locey 2005) (Fig. 3). If an exposure via (drinking) water turns out to be LTL and the estimated or measured concentration of the chemical of concern lies below the health-based drinking water limits (GLVs), no LTL risk assessment is required. Whereas, if the exposure levels exceed the GLVs for the given LTL exposure duration, LTL risk assessment can be conducted to exclude or confirm a health risk. When calculating exposure based on measured or modelled data, three exposure units can be obtained (USEPA 2003): the average daily dose (ADD) (when the contaminant is known to cause non-carcinogenic or non-chronic effects), the lifetime average daily dose (LADD) (when the contaminant is known to cause carcinogenic or chronic effects), and the acute dose rate (ADR). The difference between these three exposure units is the averaging time (AT). In the case of ADD, AT is set to the exposure duration over which exposure has occurred. In the case of LADD, the average duration is set to a lifetime even though the exposure does not occur over the entire lifetime (average 70 years). ADR is also calculated using the same equation as ADD and LADD, but AT is equal to one day. Toxicity primarily depends on peak exposure concentrations (Fig. 4), which may be concentration- or dose-driven i.e. independent of duration (but must be maintained for a minimal time) or frequency of occurrence (Haber et al. 2016). Therefore, to evaluate toxicity, it is important to determine whether the toxicity is mainly due to the concentration of the chemical during peak exposure periods or due to the total dose (i.e. concentration multiplied by time), taking into account kinetics and/or bioaccumulation. Depending on whether the concentration or the total dose is responsible for driving the adverse effect, dose averaging may be chosen over the exposure period of interest in combination with an evaluation of the peak exposures. These peaks may be evaluated by comparing them with an ARfD, if available. If no ARfD is available, a pragmatic approach may be used (expert judgement) depending on the factor by which an HBGV for longer exposure duration has been exceeded. Dose averaging requires averaging exposure periods with non-exposure periods, hence potentially underestimating the actual health risks associated with the chemical. If peak exposure is relevant or reoccurring, we advise using dose averaging in an LTL exposure scenario by considering only the peak exposures and assuming that they remain the same over the entire exposure period. In the present example (see case study), we describe the LTL exposure assessment in a fluctuating exposure scenario. The method for estimating the exposure rate is described in TEXT BOX 1 (see supplementary data S3 for details).

TEXT BOX 1. Estimation of exposure rate $\text{ADD oral}\hspace{0.17em}=\hspace{0.17em}\frac{{C}_{\text{w}} \times \text{ IR }\times \text{ EF }\times \text{ ED}}{\text{Bw }\times \text{ AT}}$

where ADD is the average daily dose (intake) [mg/kg bw-day], oral is the amount of substance consumed via ingestion, *C*_w_ is the concentration of target chemical in water [mg/L], IR is the ingestion rate of water [default intake = 2L/day adult (70 kg bw), 1L/day children (12 kg bw), EF is the exposure frequency i.e. number of exposure events over the length of time [days/week or year], ED is the exposure duration is the length of time over which exposure occurs [days or weeks or years], BW is the body weight [kg] [default BW = adult (70 kg), children (12 kg), bottle-fed infants (5 kg)], AT is the averaging time (days), the period of time over which the exposure is relevant for health risk characterization.

During exposure assessment (both chronic and LTL), whole populations can be considered, including all life stages in one calculation. Depending on the exposure scenario and relevant chemicals, consideration should also be given to specific life stages of the exposed individuals (infants, toddlers, children, and elderly). Some age groups may have higher susceptibility (e.g., the unborn and the elderly) and can thus be considered separately in the risk assessment. Attention should be paid to whether the exposure is retrospective or prospective. A retrospective risk assessment involves determining the individuals who have been exposed to the chemicals of concern already released in the environment (e.g. during an accidental release), whilst a prospective risk assessment involves determining the population that is undergoing exposure or are anticipated to be exposed in the future (PHE 2019; Borghi et al. 2020; OECD, Test No. 296). The prospective approach is typically used for regulatory purposes and can be aimed at risk mitigation and choice of risk management options (Borghi et al. 2020). In addition, several other considerations must be taken into account to estimate exposure, such as considering the likeliness of cumulative exposure (i.e. combined exposure to multiple chemicals through different exposure pathways over a period of time). This is particularly important in assessing drinking water quality, as simultaneous exposure to multiple low-level chemicals from drinking water is common (WHO 2017).

#### Step 3: hazard assessment of chemicals identified in (drinking) water in LTL scenarios

Exposure assessment is followed by a toxicological assessment to identify potential risks to human health and to set priorities for monitoring and abatement (Baken 2018). In an LTL exposure scenario, the toxicological assessment of chemicals can be conducted from the available scientific data (e.g., epidemiological, animal and in vitro studies) on chemicals of concern. If effect data are incomplete or unavailable, non-testing (in silico) approaches may be used to assess hazards. Such tools do not provide safe exposure levels but are useful for rapidly identifying potential hazards, prioritising compounds for further testing, and providing mechanistic information (Baken 2018). Non-testing tools need to be selected on a case-by-case basis to perform and evaluate hazard predictions, and multiple non-testing approaches should be combined to obtain the best prediction of toxicity (Baken and Kools 2014; Baken 2018).

#### 3a: hazard assessment of carcinogenic effects from LTL exposure

Carcinogens are substances or agents capable of causing cancer or increasing the incidence of cancer in humans or experimental animals (Schrenk 2018). Carcinogens can be classified as non-genotoxic or genotoxic based on their mode of action (MoA). Non-genotoxic carcinogens are known to induce tumours by disrupting cellular structures and by altering the rate of either cell proliferation or processes that increase the risk of genetic error (Hartwig et al. 2020; Nohmi 2018; Lee et al. 2013). Genotoxic carcinogens cause tumours by directly affecting the genetic material (e.g., DNA, chromosomes). Such substances can induce gene mutations, structural chromosome mutations and genome mutations (Nohmi 2018; Hartwig et al. 2020). If the carcinogenic MoA is not identified, then from the precautionary perspective, the carcinogenic substance is often assumed to be a genotoxic carcinogen (EFSA 2005). The threshold for a carcinogen is the exposure level below which there is no increased risk of cancer. There is a general consensus among scientists and regulators about the acceptance of an increased cancer risk (USEPA 2005). The cancer risk levels are policy decisions of health authorities and are 1-in-100,000 (≤ 1 × 10^–5^) according to WHO and 1-in-1,000,000 (≤ 1 × 10^–6^) in the Netherlands. A risk level of 1 in a million means that, among 1-million people exposed to a specific chemical concentration continuously (24 h per day) over 70 years (an assumed average lifetime), up to one individual can develop cancer. For non-genotoxic carcinogens, it is generally assumed that a threshold exists, because the mechanism leading to carcinogenesis has a lowest “effect level”. In contrast, for genotoxic carcinogens, it is generally assumed that there is no threshold and that even a very low dose can contribute to adverse effects; therefore, a “no effect level” (i.e. no observed adverse effect level or NOAEL) cannot be derived (SciCom 2018; Hartwig et al. 2020).

To assess the carcinogenic potential of a chemical, the initial step is to determine whether relevant carcinogenicity data are available. If chemical-specific data on the carcinogenicity are available, a cancer risk assessment can be performed for that compound, preferably using mechanistic and toxicokinetic data. The use of mechanistic data (e.g., the studies on the interactions of the chemical of interest with cellular macromolecules) can lead to a better selection of critical effects, identification of a susceptible population, increased confidence in hazard identification, elucidation of the relevance of animal data to humans, susceptibility, mode of action, and/or support biological plausibility linking a chemical exposure to an adverse outcome such as carcinogenicity. Toxicokinetic data which include a description of the rates of distribution of chemicals in tissues allow for better interpretation and prediction of the fate of chemicals in the body (Vandenberg et al. 2010). However, toxicity and kinetic data are often lacking (Knepper et al. 2020). In such cases, data gaps can be filled (whenever possible) using read-across and structure–activity relationships in (Q)SAR models (Felter et al. 2011). Once the available data confirm that carcinogenesis is the key concern for risk assessment, the relevant mechanisms of action, including those of any degradation or transformation products, should be considered in tumour formation. Determining whether the data supports a non-linear (non-genotoxic/threshold carcinogens) or linear dose (genotoxic/non-threshold carcinogens) response relationship dependent on the criteria set in the risk assessment requires an understanding of MoA. For illustration, the criteria to decide on linearity are provided in Table 4.

**Table 4:** Criteria for linear and non-linear MoA (adapted from: Villanueva et al. 2014; Kirkland et al. 2016)

Mode of action	Non-threshold or linear (genotoxic)		Threshold or non-linear (non-genotoxic)
Description	Cause tumours by:		Cause tumours by:
	Directly acting on DNA (these are reactive and do not require metabolic conversion)	Indirectly acting on DNA (require metabolic activation and conversion in the body to reactive chemicals)	Indirectly affecting structures of DNA or gene expression to alter chromosomal number/integrity. (e.g., peroxisome proliferators, hormones, and local irritants)
Mechanism	Interaction at a specific location or base sequence of the DNA structure causing lesions, breakage, fusion, deletion, mis-segregation or non- disjunction leading to damage and mutation		Cytotoxicity, receptor-mediated endocrine modulation, non-receptor mediated endocrine modulation, tissue-specific toxicity and inflammatory responses, immunosuppressants, or gap junction intercellular communication inhibition, altered methylation, oxidative stress
In-silico test result	Structural alert: positive		Structural alert: negative
In-vitro test result	Ames test: positive		Ames test: negative
In-vivo test result	Carcinogenic in both rats and mice and in more than one organ		Carcinogenic in single species and single organ in rodents
Some examples of chemicals in (drinking) water	Acetaldehyde, acrylonitrile, aromatic amines, azo dyes, aflatoxinb1, bromate, benzo(a)pyrene, nitrosamines, dimethylbenzanthracene, insecticides, fungicides, trichloroethylene, formaldehyde, trichloroethylene		Cadmium, diethanolamine, dieldrin

**Fig. 5:**
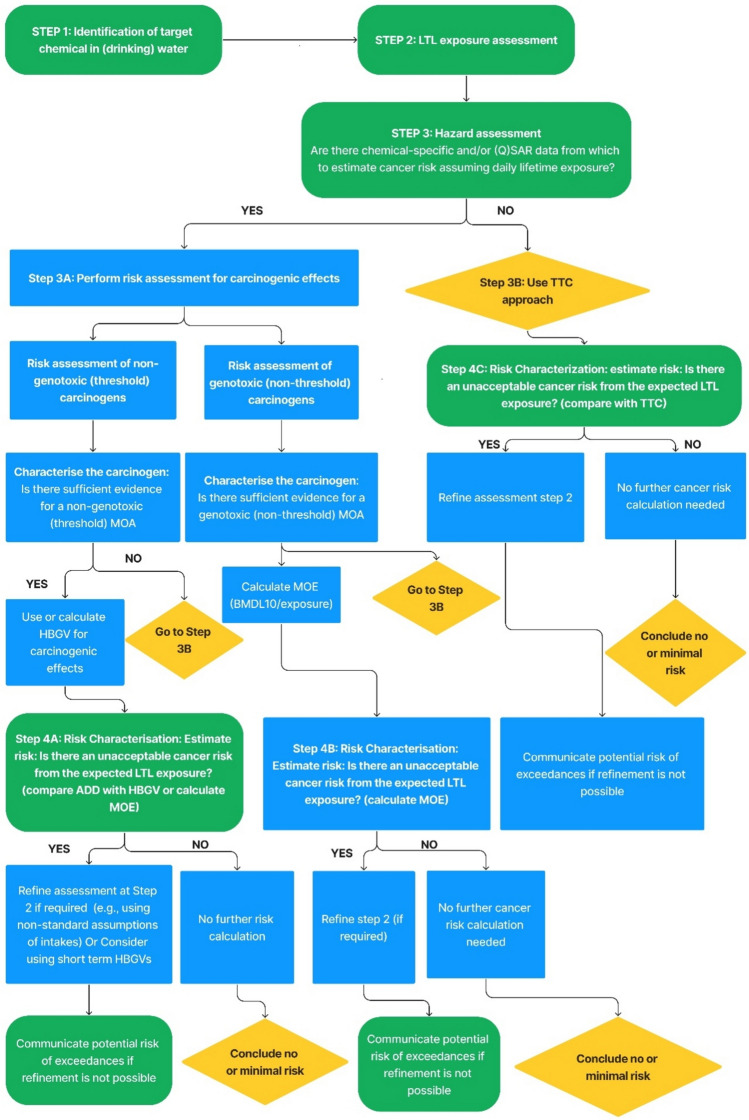
Flowchart for assessing risk from LTL exposure to carcinogenic chemicals in (drinking) water. The scheme is based on the framework proposed by Felter et al (2011) for assessing risks from LTL exposures to carcinogens, combined with the principles developed by Public Health England (PHE 2019). Green = Start and end of steps; Blue = Process; Yellow = Decision (color figure online)

When a specific MoA is established, the workflow described under Step 3A in the workflow should be followed (see Fig. 5). Non-genotoxic carcinogen risk assessment should involve deriving an HBGV and applying suitable uncertainty factors (UFs). If a lifetime HBGV exists for the carcinogenic effect, it should be used; otherwise, an HBGV should be established to align with the LTL exposure period. The preferred point-of-departure (POD) for the HBGV is the benchmark dose (BMDL_10_). However, if BMDL_10_ is not available, the NOAEL [or Lowest-observed-adverse-effect level (LOAEL)] with appropriate UF may be used. Selected UFs should account for differences in toxicokinetics and toxicodynamics between animals and humans and among human populations. Default UFs may vary due to interindividual variability of population sub-groups (pregnant women and their foetuses, new-borns, young children, or elderly). If data are insufficient to establish an HBGV, a margin of exposure (MOE) approach may be used based on the most appropriate POD and taking uncertainty into account. The MOE is the ratio of POD (preferably the BMDL_10_) obtained from animal toxicology studies to the predicted and/or estimated human exposure level. It should be noted that the use of HGBV or MOE based on long-term toxicity studies may be considered precautionary when applied to short-term LTL scenarios. If an LTL exposure scenario being evaluated indicates a higher exposure than the HBGV, refining risk assessment may be considered appropriate, such as visualising uncertainty in the exposure and toxicity data (e.g., using Risk21 software). In addition, a short-term study can be used to establish a more appropriate HBGV for LTL exposures. There are often mixed MoAs, as well as dose-dependent transitions in MoA, which contribute to the complexity of understanding the potential for a linear or nonlinear dose–response in the low-dose range (Slikker et al 2004; Felter et al. 2011). Although some regulatory agencies assume of a “linear, no threshold” model for mutagenic carcinogens, this assumption is increasingly being challenged, as studies indicate nonlinearity of mutagenic compounds (Lutz 2009). While scientific developments are followed, the formal guidance by competent authorities are leading for the framework presented here.

If a cancer risk cannot be estimated for the chemical, then a default approach such as the threshold of toxicological concern (TTC) may be considered (Baken and Sjerps 2016). The TTC is a science-based, practical approach for evaluating low-level chemical exposures when chemical-specific toxicity data are limited or unknown (EFSA 2019; Ellison et al. 2021). It was developed to prioritise risk assessment of substances with known chemical structures, including those with or without structural alerts for genotoxicity as well as cancer and non-cancer endpoints. This approach is particularly useful when human oral exposure is expected to be low. If exposure exceeds the relevant TTC value, further evaluation on a case-by-case basis is recommended. Some authors propose that TTC values should be higher for LTL exposures than for chronic exposures, particularly in pharmaceutical contaminants, cosmetics, and trace chemicals with structural alerts for genotoxicity (EMA 2006; Müller et al. 2006; Kroes et al. 2007; Felter et al. 2009, 2011). An expert workshop emphasised the need for a dedicated database on acute and other LTL toxicity to establish LTL-specific TTC values (EFSA and WHO 2016). Such values can play a significant role in LTL risk assessment. If exposure to a chemical is below the TTC value, the chemical can be considered as having a low probability of risk with reasonable confidence (Munro et al. 1996; Ellison et al. 2021). TTC levels and corresponding TTC-based drinking water guideline values are given in Table 5 (Baken 2018).

**Table 5:** TTC levels and TTC based drinking-water guideline values (Source: Baken 2018)

Classification	TTC (µg/day)	References	TTC-based drinking water target value (µg/L)	References
Cramer class I (low toxicity)	1800	Munro et al. (1996)	37.7	Baken and Sjerps (2016)
Cramer class II (medium toxicity)	540	Munro et al. (1996)	–	
Cramer class III (high toxicity)	90	Munro et al. (1996)	4.0	Baken and Sjerps (2016)
Organophosphates and carbamates	18	Kroes et al. (2004)	–	
Carcinogens	1.5	TOR rule (’80)	0.1	Mons et al. (2013)
Genotoxic substances (except aflatoxins, azoxy- or N-nitroso compounds	0.15	Kroes et al. (2004)	0.01 0.02	Mons et al., (2013) Baken and Sjerps (2016)

(–) No values were calculated. The threshold values were only derived for compounds that were relevant to drinking water. These substances mostly shared structural similarity with Cramer classes I and III and genotoxic compounds

#### 3b: hazard assessment of non-carcinogenic chemicals from LTL exposure

**Fig. 6:**
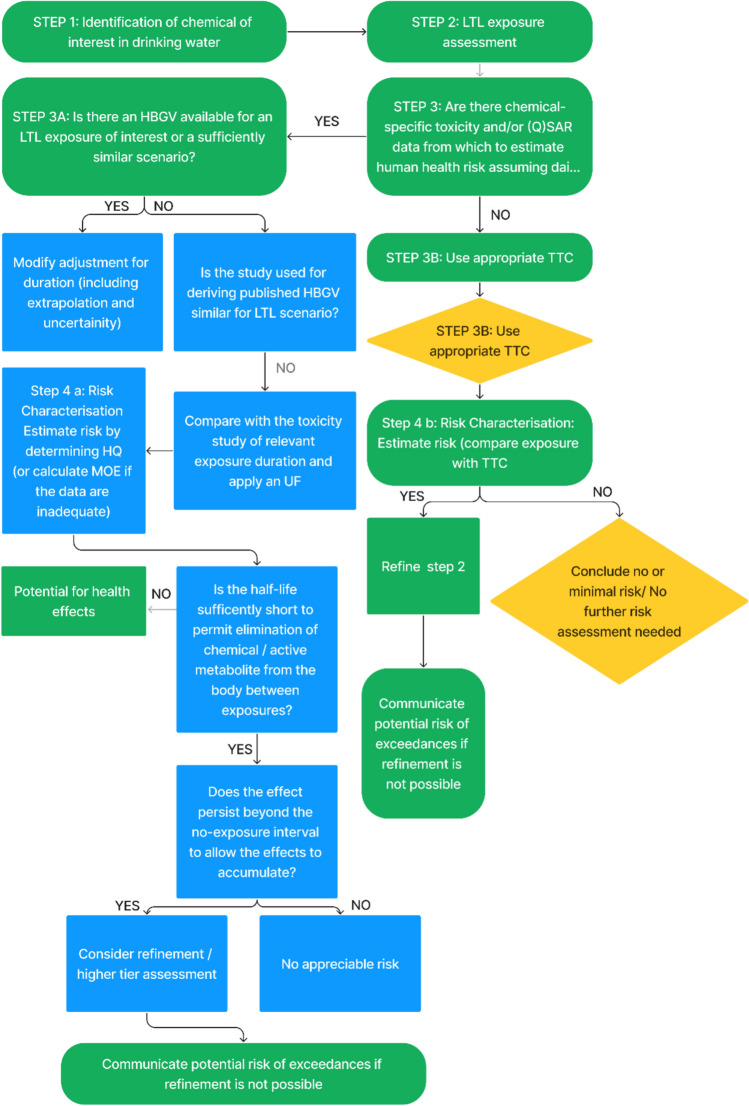
Flowchart for assessing risk from LTL exposure to non-carcinogenic chemicals in (drinking) water. The scheme is modified from Haber et al. (2016). Green = Start and end of steps; Blue = Process; Yellow = Decision (color figure online)

The initial step in evaluating the non-carcinogenic effects of LTL exposure involves identifying any existing acute or sub-chronic chemical-specific toxicity data/or QSAR data that can be used to estimate human health risks. If such data are available, the assessment should proceed with Step 3A of the workflow (see Fig. 6). At this stage, it is essential to determine whether a reference value (RfV) [health-based guidance value (HBGV)] exists for the given exposure scenario. If a relevant RfV is unavailable, the next consideration is whether the study used to establish the RfV is similar in duration to the exposure scenario being assessed. If the study duration aligns with the exposure scenario, the RfV may be adjusted by modifying or removing UFs that were initially applied for extrapolation purposes. For example, if an RfV included a UF to account for extrapolation from sub-chronic to chronic exposure, and the current assessment pertains only to sub-chronic exposure, the UF for this extrapolation can be removed. If no directly applicable study exists, the next step is to compare the available toxicity studies with relevant exposure durations and apply appropriate uncertainty factors. When modifying an RfV by eliminating a UF, it is crucial to reassess whether a different toxicological endpoint might become a critical concern for the given duration of exposure.

In cases where no chemical-specific toxicity or QSAR data are available, Step 3B of the workflow should be followed. This step requires determining whether exposure assessments should rely solely on short-term reference values or if long-term values should also be considered. The toxicokinetic and toxicodynamic properties of the chemical in question play a key role in this determination. Specifically, two factors should be evaluated: (1) whether the chemical or its degradation product remains in the body due to low elimination relative to the interval between exposure episodes, and (2) whether the chemical exhibits sufficient metabolic persistence to accumulate over multiple exposures (Haber et al. 2016). If either of these conditions is met, the exposure must be assessed against a reference value applicable to the entire exposure duration. To estimate the potential for accumulation, the chemical’s elimination half-life or that of its active metabolite can serve as a key metric. As outlined by Haber et al. (2016), a chemical is considered non-accumulating if the interval between exposure episodes is at least five times its elimination half-life, provided that elimination mechanisms are not saturated. If elimination follows a biphasic or multiphasic pattern where an initial rapid elimination phase is followed by a prolonged terminal phase, the longer half-life should be used as a precaution unless there is sufficient justification for using the shorter one. In specific cases, such as when a chemical is stored in fat or bone and only released under certain conditions, using the shorter half-life may be appropriate. Another critical aspect of intermittent exposure is determining whether the effects of exposure persist between episodes. This involves assessing whether non-adverse biological changes could progress with repeated exposure, even at concentrations below established thresholds for adverse effects (Haber et al. 2016). In addition, it is important to consider not just whether exposure levels exceed the RfV but also by how much, as this can influence the overall risk assessment.

#### Step 4: risk characterisation

Risk characterisation is the final stage of the risk assessment process and integrates information from the exposure and hazard assessment process and includes a synopsis and synthesis of all the data that should contribute to a conclusion about the nature and extent of the chemical-induced risk in an LTL exposure scenario, i.e. whether adverse effects can be expected from the given LTL exposure (carcinogenic or non-carcinogenic effects). For non-carcinogenic chemicals a risk can be characterised by comparing estimated (or measured) exposure (ADD) with the available non-cancer health guidelines such as RfDs, and this is called Hazard Quotient (HQ) (TEXT BOX 2; see supplementary data S3 for details). In case there are inadequate data, MOE should be calculated. For carcinogenic effects, risk characterisation depends on the mechanisms of carcinogenicity and the relationship between dose and carcinogenic response. Different approaches have been used to characterise the risk for toxic effects that are considered to have a threshold or non-threshold (TEXT BOX 3; see supplementary data, S3 for details). For genotoxic effects, it is generally assumed that there may be no threshold and that there is some risk at any level of exposure. Therefore, for substances that are genotoxic and carcinogenic, exposure levels should be controlled to as low as reasonably achievable (ALARA) (PHE 2019). It should be noted, however, that some chemicals increase the incidence of cancer in experimental animals by non-genotoxic mechanisms, and HBGV would be appropriate for such chemicals.

TEXT BOX 2: Risk characterisation for non-carcinogens (a) HQ = ADD/RfD or MRL,

where HQ is the hazard quotient, RfD is the reference dose [mg/kg/day], MRL is the minimal risk level [mg/kg/day].

HQ > 1, implies significant non-carcinogenic health risk,

HQ ≤ 1 implies that exposure may not lead to non-carcinogenic health risk

TEXT BOX 3: Risk characterisation for carcinogens (a) Calculating lifetime cancer risk associated with LTL exposures

$$\text{ILCR}\hspace{0.17em}=\hspace{0.17em}\frac{D \times \text{ CSF }\times \text{ ADAF }\times \text{ ED}}{\text{LT}}$$

ILCR is the incremental lifetime cancer risk*, *D* is the exposure dose [mg/kg bw-day], CSF is the cancer slope factor [mg/kg-day], ED is the exposure duration [years], ADAF is the age dependent adjustment factors (10 for children 0 < 2 years; 3 for children to < 16 years; = 1 for children ≥ 16 and adults, LT is the lifetime [years] (considering 70 years average expectancy).

*A quantitative estimate used in risk assessment to evaluate the probability of an individual to develop cancer over their lifetime due to exposure to a carcinogenic substance.

### Practical applications of LTL framework

The decision-tree framework for assessing LTL exposures can be applied in different scenarios in practise. For example, in an agricultural runoff scenario, the framework can be used to identify specific pesticides used in nearby fields, monitor water samples during peak planting seasons, and assess how exposure duration and/or frequency of these pesticides affect the population, particularly the vulnerable populations such as children. Similarly, in the context of industrial discharges, the framework can help characterise the potential health risks to the local community. This can be particularly valuable when the emissions vary over time, such as in seasonal patterns or during operational changes. Furthermore, the framework can be effective in cases of natural contaminants, like rising chemical levels due to seasonal flooding or urban runoff after heavy rainfall. In these scenarios, the framework can track how contaminant levels fluctuate and assess whether these temporary spikes in exposure, despite their short duration, pose a risk to public health, particularly for sensitive groups.

## Results: lead case study

Low-level lead (Pb) exposure has long been associated with adverse health effects, particularly in children. However, health concerns in adults also exist (ATSDR 2020). According to the latest technical report by WHO, a proactive approach should be taken to identify, assess and manage Pb in drinking water (WHO 2022a, b, c). This involves identifying Pb sources in drinking water, monitoring Pb levels in water supplies (particularly those known or suspected to contain lead materials) and implementing appropriate procurement and installation procedures to prevent Pb contamination in new water systems. Lead contamination of drinking water is a problem of public concern, and regulators across the world have focussed on taking actions in the interest of public health (ATSDR 2020; Lim et al. 2012). Given that Pb concentrations in drinking water can fluctuate depending on water use patterns and distribution system dynamics, the framework for the LTL risk assessment of chemicals in (drinking) water is illustrated with a case study on modelled fluctuating concentrations of Pb in drinking water.

### Identification of chemicals in (drinking) water (step 1)

Tap water from old lead water pipes in the premise plumbing (in-house installation) accounts for a large portion of total daily Pb exposure of the people living in these houses. Even in newly built homes with new plumbing and faucets, brass fittings can lead to undesired concentrations of Pb in drinking water, which is the most prominent in the first few months of use (Elfland et al. 2010a, b). To assess whether a consumer is exposed to undesirable amounts of Pb, sampling protocols play a crucial role. Sampling protocols appear in various forms, and each protocol typically has its own objective (for example, to address Pb exposure at a community scale, Pb exposure at a household scale, presence of Pb-releasing components, or the localisation of Pb-releasing components). To test the effectiveness of the various sampling protocols in a controlled environment, simulations of a premise plumbing system were performed using EPANET (Bonora et al. 2022). EPANET is a software tool designed to model water distribution systems and analyse the transport and behaviour of drinking water and its micropollutants in these systems. In this case, it was used to model domestic drinking water system. Advantages of this method (modelling) include control and knowledge of the location of Pb-releasing components and Pb dissolution behaviour, the ability to control water demand patterns, as well as the ability to accurately measure lead exposure (Dash et al. 2022). It should be noted that the modelling has its own simplifications, and some aspects that occur in reality (such as the occurrence of particulate Pb) is missing in the simulations.

### Exposure assessment (step 2)

Data for the exposure assessment were obtained by modelling the flow of dissolved Pb in water using EPANET (Dash et al. 2022). In this case, a typical (fictitious) Dutch house with water usage spread over three floors was considered, and the Pb exposure in an average family with two children was estimated (Dash et al. 2022). The starting point of the simulations was to define the indoor plumbing system. For this purpose, the lengths and diameters of the pipes as well as the points of use were defined (see supplementary data: S1). Information on the point of use, and composition of the household (number of adults and children, attitude towards water usage) was fed into SIMDEUM, which is a stochastic drinking water demand model (Blokker et al. 2017). SIMDEUM generates water demand patterns for various points of use based on the input information. The output from SIMDEUM is coupled back to the EPANET model. The leaching of dissolved Pb from various pipes was modelled.

Using the modelling framework, water consumption was evaluated for a household on a weekly basis for a total period of 20 weeks. In the simulation considered for Pb exposure, Pb was assumed to leach from the pipes (in total one metre long and 32 mm in diameter). It was assumed that the concentration of Pb in water from the distribution network is undetectably low (e.g., < 0.2 µg/L). The plumbosolvency (Pb concentration at equilibrium) was assumed to be 110 µg/L, whereas the Pb dissolution rate was assumed to be 0.115 µg/(m^2^s). These values are known to be dependent on parameters such as water chemistry and temperature (KIWA, 1986). However, these factors were considered as constants and not considered further in this study. Modelled Pb levels in tap water varied (fluctuated) between 2.4 and 5.4 µg/L (average 3.8 µg/L) (fluctuating exposure, see Fig. 1) over a period of 20 weeks at the kitchen tap (for cold water). The average Pb concentration computed using the modelling framework includes all events (drinking water, cooking food, rinsing dishes, washing hands etc.) and no further disaggregation has been performed. We applied a conservative approach and used the peak concentration of 5.4 µg/L (see supplementary data: S2), assuming that the concentration remains the same throughout 20 weeks. Exposure was estimated for two groups: children (at extra risk) and adults (as the general population), following the USEPA method and the publication of Alidadi et al. (2019) (USEPA 2011) with some modifications [e.g., the default body weight is based on the value selected by EFSA (2012)]. The differences in the exposure doses between children and adults are based on the difference in their body weight (see Table 6).

**Table 6:** Risk characterisation in two age groups based on non-cancer and cancer effects

Exposure duration	Population	Exposure type	Exposure dose	Non-cancer effects		Cancer effects	
	Age group		(mg/kg/bw-day)	HQ	Risk	ILCR	Risk
LTL	Adults	ADD_oral_	1.5 × 10^–4^	< 1	acceptable	6.99 × 10^–6^	unacceptable
	Children	ADD_oral_	4.5 × 10^–4^	< 1	acceptable	6.29 × 10^–5^	unacceptable

*LTL* less than lifetime, *ADD* average daily dose, *HQ* hazard quotient (calculated only for non-cancer effects), *ILCR* incremental lifetime cancer risk

### Hazard assessment (step 3)

The adverse effects of Pb are well known, and it is probably the most extensively studied heavy metal. Studies have reported the presence of various cellular, intracellular, and molecular mechanisms behind the toxicological manifestations of Pb in the body (Gillis et al. 2012; Shvachiy et al. 2022; Virgolini and Aschner 2021). Pb is known to induce neurological, respiratory, urinary, and cardiovascular disorders due to immune-modulation, oxidative, and inflammatory mechanisms (ATSDR 2020; Balali-Mood et al. 2021). Childhood exposure to Pb is associated with long-term decreases in intelligence quotients (IQs) (Halabicky et al. 2022). Emerging evidence shows the potential role of the gut microbiota in mediating some of these effects, particularly through the microbiota–gut–brain (MGB) axis (Duan et al. 2023). Pb exposure can alter gut microbiota composition, leading to dysbiosis, which is characterised by reduced microbial diversity and the overgrowth of pathogenic bacteria, such as certain strains of *Clostridia*, depletion of beneficial bacteria like *Lactobacillu*s and *Bifidobacterium* (Wang et al. 2018; Kamer et al. 2024). Dysbiosis can compromise intestinal barrier integrity, increasing gut permeability and enable the translocation of bacterial products into the bloodstream, which may trigger systemic inflammation (Fine et al. 2019). Neuroinflammation, a key consequence of these processes, plays a central role in mediating effects of Pb on cognitive and behavioural outcomes, e.g., by overactivation of microglia, the immune cells of the brain (Tizabi et al. 2023).

The evidence for the carcinogenicity of Pb in humans is inconclusive because of the limited number of studies and the failure to adequately account for potential confounding variables. Previously, Pb and inorganic Pb compounds were classified in Group 2B (possibly carcinogenic to humans), whereas organic Pb compounds were classified in Group 3 (not classifiable as to its carcinogenicity to humans). The most recent IARC evaluation resulted in a revision. Inorganic Pb compounds are now included in Group 2A (probably carcinogenic to humans), whereas organic Pb compounds remain in Group 3 (IARC 2022; Rousseau et al. 2005; WHO 2016). However, the IARC Working Group noted that some of the organic Pb is metabolised into ionic Pb, which is expected to have the same toxicity as inorganic Pb (Rousseau et al. 2005). Based on the Drinking Water Directive (DIRECTIVE (EU) 2020/2184, EU, 2020) and the World Health Organisation (WHO 2016), the permissible limit of Pb in drinking water is set as 10 μg/L. It should be noted that the guidance value (GV) of Pb (10 µg/L) is designated as provisional on the basis of treatment performance and analytical achievability and is no longer health-based. Concentrations are required to be maintained as ALARA, with the objective of meeting a target value of 5 µg/L by January 2036 (WHO 2016). However, Pb is not an essential element (elements that are necessary or beneficial for health), and there is no safe level of Pb exposure (Flora et al. 2012; Vorvolakos et al. 2016; WHO 2022a, b, c).

### Risk characterisation (step 4)

Here we estimate both the non-cancer risk of Pb based on the ADD calculated from the (modelled) exposure data and the cancer risk (as Pb is classified as Group 2A by IARC). We used hazard quotients (HQ) to estimate the non-cancer health risk of Pb in (drinking) water (see supplementary data: S3). The calculation for HQ requires information on the exposure concentration of Pb in drinking water and the RfD for Pb that is likely to be without an appreciable risk of harmful effects during a lifetime exposure. Exposure concentrations were obtained by modelling the flow of dissolved Pb in drinking water (sampled at the tap). In recent assessments, the authorities did not recognise a safe level for Pb exposure, therefore, there is no recommended reference dose (RfD)/health-based value for Pb. However, different oral RfDs have been reported in the literature (1.4 × 10^−3^ to 4 × 10^−3^ mg/kg bw-day) (Aendo et al. 2019; Guo et al. 2018a, b; Nag and Cummins 2022). In our study, the HQ for Pb was calculated based on the lowest (precautionary) RfD of 1.4 × 10^−3^ mg/kg bw-day (1.4 µg/kg bw-day). To estimate the carcinogenic health risk of Pb in (drinking) water, we used the oral cancer slope factor for Pb (see supplementary data: S3). EPA has not developed an oral slope factor for lead because of the many uncertainties, some of which may be unique to Pb. We therefore used the oral slope factor of 0.0085 mg/kg-day derived by the Office of Environmental Health Hazard Assessment (OEHHA), California (OEHHA 2023a, b). The results obtained based on the measured exposure concentrations (adults: 0.15 µg per kg bw-day, children: 0.45 µg per kg bw-day) and the oral CSF were compared with the already established risk limit of 1-in-1,000,000 (≤ 1 × 10^–6^) (van der Aa et al. 2017). The results showed unacceptable cancer risk in adults in an assumed short-term exposure scenario of 20 weeks in both age groups. Furthermore, the risk was higher in children.

## Discussion

A method for LTL risk assessment allows characterising risks of realistic chemical exposure scenarios that represent exposure over (very) short or intermediate periods. A modelled 20-week exposure to Pb in a typical Dutch household was used as a case study to illustrate the application of the proposed method for LTL risk assessment of (drinking) water. This is relevant because in some parts of the Netherlands and beyond, houses (and buildings) contain Pb pipes, and consequently there is a risk of leaching of Pb from Pb-containing components/materials (WHO 2022a, b, c). This case study assumed that no Pb exposure occurs before or after the 20-week period. It must also be noted that this case study is based on a worst-case scenario in which the maximum modelled concentration of Pb in the water was used in the risk characterisation. The case study was used to illustrate the method and was not intended for comprehensive risk assessment (i.e. considering variability and uncertainties associated with spatiotemporal contexts, population-specific behaviours, age-specific anthropometrics, vulnerable sub-population groups). However, in a real-life scenario, if an unacceptable risk is identified, it is recommended to refine the risk assessment to reach a final conclusion on the risk. This could include more comprehensive exposure assessment that takes into account various factors, such as the internal body concentration variability and uncertainties associated with spatial and temporal context, population-specific factors, and vulnerable populations. Probabilistic risk assessment (PRA) can play a key role in addressing these uncertainties and a realistic understanding of the potential risks associated with water contamination. PRA characterizes uncertainties by considering variability in input parameters (e.g., body weights, water consumption rates, exposure durations) and as well as model assumptions. This allows for the generation of a range of possible risk outcomes, providing a more detailed understanding of the risks. A method for PRA specifically tailored to assessing drinking water quality is presented in our report which will be public in April 2025 (KWR 2024).

One of the strengths of this case study was the innovative exposure assessment using a modelling framework in a typical (fictitious) Dutch house via drinking water. An added value of our modelling approach is that it shows the concentrations during showering and could thus also serve to determine the exposure through inhalation of aerosols or dermal contact (Dash et al. 2022). The concentration of Pb in (drinking) water depends on the type of material, general water chemistry, and temperature. However, these factors were not considered in this study and can be addressed in future research. In addition, the calculated Pb exposure includes all events (drinking water, cooking food, washing dishes, washing hands, etc.) undertaken at all taps in the modelled house. No further disaggregation was made but this can be addressed in future research, in which the contribution of oral and dermal exposure can be addressed. Furthermore, an important criterion to be considered is the time of exposure (e.g., if exposure in the morning is higher than in the afternoon). The WHO currently recommends a parametric value of 10 μg/L (or as low as reasonably practicable), whilst in the future (by 12 January 2036), a stricter standard of 5 μg/L is required to be met (Directive (EU) 2020/2184). The modelling framework presented in the case study can thus also be of interest to water utilities and health authorities that want to know if copper pipes with Pb solder and/or brass components or any specific components in the in-house distribution system can lead to the exceedance of the forthcoming stricter standards.

In the present case study, the health risk was estimated based on a risk threshold, although it is generally assumed that there is no safe threshold for Pb exposure (Vorvolakos et al. 2016). HQ was used to characterise the non-carcinogenic risk of Pb at a modelled (drinking) water concentration of 5.4 µg/L and a duration of 20 weeks. HQ was lower in adults (0.11) than in children (0.32). Nevertheless, both values are lower than the safe level of 1, indicating that potential human health effects at this concentration and duration of exposure are not expected. The difference in the HQ is assumed to be due mainly to the difference in body weight, which is 70 kg for adults and 12 kg for children. The cancer risks estimated in the present study were all above the acceptable level of 1 × 10^–6^ in both population groups (van der Aa et al. 2017). Compared to the adults, the modelled risk was higher in children. In the case study, a maximum measured concentration of Pb in treated water is used for characterising its risk to human health, under a worst-case scenario approach. While this approach is methodologically sound, it should be emphasised that this also represents an extreme and unlikely exposure scenario. The health risk assessment for the carcinogenic and non-carcinogenic effects is also not considered adequate to allow calculation of a (LTL) GLV for Pb in drinking water. The HQ values only provide an indication of the levels at which adverse health risks may be considered when evaluating lead exposure in drinking water for LTL scenarios. However, expanding modelling to encompass additional exposure scenarios can provide more insight into possible health risks. Based on the assessment, management decisions and/or mitigatory measures can be taken.

Overall, our results show LTL risk assessment as valuable tool for evaluating the potential short-term presence of chemicals in water intended for human consumption. A critical aspect of this assessment is the incorporation of acute and sub-chronic effects, which is essential for a better understanding of chemical exposure risks. To effectively integrate these effects into the risk assessment framework, it is crucial to utilise HBGVs specifically designed for acute and sub-chronic exposures. This includes establishing short-term exposure limits that consider high-concentration scenarios and evaluate cumulative exposure risks from intermittent exposure to chemicals. Based on the results of the LTL risk assessment, risk mitigation strategies can be implemented, such as avoiding the use of water distribution components that may contribute to exposure and selecting materials that comply with the minimum requirements for substances in contact with drinking water, as outlined in the Drinking Water Directive (Directive (EU) 2020/2184).

## Conclusion

This study presents a decision tree framework for assessing LTL exposures to chemical contaminants to address the challenges posed by fluctuating contaminant levels in drinking water. The framework enables a better evaluation of potential health risks in cases where contaminant concentrations vary over time. This structured approach is demonstrated using a case study on Pb, which provides risk assessors and water quality managers with a practical tool for characterising short-term exposures. Several aspects require further attention. A significant obstacle is the lack of comprehensive data on time-varying concentrations of many contaminants in drinking water and their corresponding health impacts. Limited toxicological data, particularly for acute and sub-chronic exposure scenarios, further complicates the risk assessment process. In addition, the framework does not yet account for the combined effects of chemical mixtures, which often represent real-life exposure scenarios. Future research should focus on refining the proposed framework to incorporate mixture risk assessment and account for cumulative and synergistic effects of co-occurring chemicals. Developing short-term HBGVs tailored to LTL exposure durations is another critical need. Advances in analytical techniques, such as non-target screening and improved modelling tools, can support more accurate exposure and hazard assessments. Furthermore, efforts to address uncertainties in bioaccumulation and toxicokinetics for intermittent exposures will enhance the applicability of the framework. Another key area for exploration is the integration of this framework into regulatory practices and decision-making processes. Customisable approaches, such as using adaptive monitoring strategies and modelling tools, can aid water utilities and regulators in responding proactively to emerging contaminants.

## Supplementary Information

Below is the link to the electronic supplementary material.

Supplementary file1 (XLSX 309 KB)

Supplementary file2 (XLSX 21 KB)

Supplementary file3 (PDF 268 KB)

## Data Availability

Data analyzed during this study are included in the supplementary information.
