# Supplementary material for: A framework for evaluating less-than-lifetime exposures: advancing toxicological risk assessment for drinking water quality
Source: Arch Toxicol. 2025 May 25;99(8):3147–68. doi: 10.1007/s00204-025-04061-9 (PMC12367986; doi:10.1007/s00204-025-04061-9)
Supplement: Supplementary file 3 — Supplementary file3 (PDF 268 KB) [file 204_2025_4061_MOESM3_ESM.pdf]

A Framework for Evaluating Less-Than-Lifetime Exposures: Advancing Toxicological Risk Assessment for Drinking Water Quality

Sanah Majid[1], Astrid Reus[1], Renske Hoondert[1], Mirjam Blokker[1], Amitosh Dash[1], Corine Houtman[2,3], Merijn Schriks[4], Milou M.L. Dingemans[1,5*]

[1] KWR Water Research Institute, Nieuwegein, The Netherlands
[2] Het Waterlaboratorium, Haarlem, The Netherlands
[3]: Amsterdam Institute for Life and Environment (A-LIFE), Vrije Universiteit Amsterdam, The Netherlands
[4] Vitens NV, Water Company, Zwolle, The Netherlands
[5] Institute for Risk Assessment Sciences, Utrecht, The Netherlands

Supplementary Data S3

TEXT BOX 1

The Averaging Time (AT), used in calculating ADD is usually different for non-carcinogenic and carcinogenic risk estimates. For non-carcinogenic chemicals, the average exposure during the contact with a chemical is generally the relevant exposure duration for risk assessment [e.g., AT = Exposure Duration (ED) * Exposure Frequency (EF)]. When actual dose is being calculated, for example in case of acute exposure AT = ED (e.g., one day) (USEPA, 2003). For cancer risk assessment, the average duration is set to a lifetime, which is usually assumed to be 70 years (70 x 365 = 25,550 days) in risk assessments (WHO/IPCS, 2021). The difference is based on the different mechanisms for cancer and non-cancer effects. For cancer, it is based on the idea that all exposures add up and manifest any time in lifetime. For non-cancer effects recovery may occur in between exposure periods in case of intermittent exposures hence the doses of multiple exposure periods cannot be added up.

$$\textbf{ADD}_{\textbf{oral}} = \frac{\mathbf{C_W \times IR \times EF \times ED}}{\mathbf{Bw \times AT}}$$

Where:

ADD = *Average daily dose (intake) [mg/kg bw-day]*
Oral = *amount of substance consumed via ingestion*
C_w = *Concentration of target chemical in water [mg/L]*
IR = *Ingestion rate of water [default intake = 2L/day adult (70 kg bw), 1L/day children (12 kg bw)*
EF = *Exposure frequency i.e. number of exposure events over the length of time [days/week or year]*
ED = *Exposure duration is the length of time over which exposure occurs [days or weeks or years]*
BW = *Body weight [kg] [default BW = adult (70 kg), children (12 kg), bottle-fed infants (5 kg)]*
AT = *Averaging time (days) is the period of time over which the exposure is relevant for health risk characterization*

TEXT BOX 2: Risk characterisation for non-carcinogens

(a) HQ = ADD / RfD or MRL

Where:
HQ = Hazard quotient
RfD = Reference Dose [mg/kg/day]
MRL = Minimal Risk Level [mg/kg/day]

HQ>1, implies significant non-carcinogenic health risk,
HQ≤ 1 implies that exposure may not lead to non-carcinogenic health risk

Or

(b) Calculate MOE if the data are inadequate

MOE = BMD10 (or NOAEL)/Estimated exposure dose (ADD)
MOE ≥ 100 is generally considered to be protective

Or

(c) Use TTC in absence of chemical specific data

ADD > TTC: risk unacceptable

ADD ≤ TTC: risk acceptable

TEXT BOX 3: Risk characterisation for carcinogens

(a) Calculating lifetime cancer risk associated with less than lifetime (LTL) exposures

$$\text{ILCR} = \frac{\text{D x CSF x ADAF x ED}}{\text{LT}}$$

D *Exposure dose [mg/kg bw-day]*

CSF *Cancer Slope Factor [mg/kg-day]*

ED *Exposure duration [years]*

LT *Lifetime [years] (considering 70 years average expectancy)*

ADAF *Age dependent adjustment factors (10 for children 0 < 2 years; 3 for children to < 16 years; 1 for children ≥ 16 & adults.*

Note: To obtain the overall risk for a 70-year period (initiated at birth), the risk is calculated for each age group and exposure periods and then added together to obtain the total. It is also possible to compute cancer risk can also be calculated for any exposure duration combined with the doses for the target age group (USEPA, 2011). In the present study, only one age group i.e. < 16 years is selected for demonstration.

Note: Cancer risks will be considered negligible where the estimated cancer risk is 1-in-100,000 ($\leq 1 \times 10^{-5}$) (WHO, 2022) or 1-in-1,000,000 ($\leq 1 \times 10^{-6}$) (van der Aa et al., 2017). The risk exceeding 1-in-10,000 ($\geq 1 \times 10^{-4}$) is considered unacceptable (USEPA, 1992; Nag & Cummin, 2022).

$\geq 1 \times 10^{-3}$: High risk

$\geq 1 \times 10^{-4}$ but $<1 \times 10^{-3}$: Moderate risk

$\geq 1\text{x}10^{-6}$ but $<1 \times 10^{-4}$:Low risk

$<1 \times 10^{-6}$: Very low risk

(b) Calculate MOE if data are inadequate

MOE = BMD10/ Estimated exposure dose (ADD)

MOE banding

MOE > 1,000,000 : highly unlikely to be a concern

MOE 10,000 - 1,000,000 : unlikely to be a concern

MOE < 10,000 : may be a concern

(c) *Use TTC in absence of chemical specific data*

ADD > TTC: risk unacceptable

ADD ≤ TTC: risk acceptable

TEXT BOX 4

For carcinogenic chemicals, the US Environmental Protection Agency (USEPA) has an oral slope factor (or an inhalation unit risk). Oral slope factor can be used for oral exposure of chemicals, for example via drinking water (or food) to calculate an incremental (increased) Lifetime Cancer Risk (ILCR) that may occur from the LADD. This number can tell the cancer risk above the existing likelihood of developing cancer. The cancer slope factor (CSF) converts estimated daily intake averaged over a lifetime of exposure directly to incremental risk of an individual developing cancer (USEPA, 1989).

References

- Nag, R., & Cummins, E. (2022). Human health risk assessment of lead (Pb) through the environmental-food pathway. Science of the Total Environment, 810, 151168. https://doi.org/10.1016/j.scitotenv.2021.151168
- USEPA (1989). Risk Assessment Guidance for Superfund, Volume I:Human Health Evaluation Manual; Office of Emergency and Remedial Response: Washington, DC, USA.
- USEPA (1992). Dermal Exposure Assessment: Principles and Applications. DERMAL EXPOSURE ASSESSMENT: PRINCIPLES AND APPLICATIONS | Risk Assessment Portal | US EPA
- USEPA (2011). Exposure assessment handbook. EPA-600-R-090-052F, Exposure Factors Handbook, 2011 Edition. (nrc.gov)
- Van der Aa, NGFM., van Leerdam, R.C., van der van et al. (2017). Evaluatie signaleringsparameter nieuwe stoffen drinkwaterbeleid. RIVM Rapport 2017-0091. Rijksinstituut voor Volksgezondheid en Milieu (RIVM). DOI 10.21945/RIVM-2017-0091
- WHO (2022). Lead in drinking-water. Health risks, monitoring and corrective actions. Technical brief. Lead in drinking-water: Health risks, monitoring and corrective actions (who.int) 2 Sep 22053 Lead in drinking-waters Technical brief (who.int)
